# Root Effect Haemoglobins in Fish May Greatly Enhance General Oxygen Delivery Relative to Other Vertebrates

**DOI:** 10.1371/journal.pone.0139477

**Published:** 2015-10-05

**Authors:** Jodie L. Rummer, Colin J. Brauner

**Affiliations:** 1 Department of Zoology, University of British Columbia, 6270 University Blvd., Vancouver, BC, V6T 1Z4 Canada; 2 ARC Centre of Excellence for Coral Reef Studies, James Cook University, Townsville, QLD 4811 Australia; Institut National de la Recherche Agronomique (INRA), FRANCE

## Abstract

The teleost fishes represent over half of all extant vertebrates; they occupy nearly every body of water and in doing so, occupy a diverse array of environmental conditions. We propose that their success is related to a unique oxygen (O_2_) transport system involving their extremely pH-sensitive haemoglobin (Hb). A reduction in pH reduces both Hb-O_2_ affinity (Bohr effect) and carrying capacity (Root effect). This, combined with a large arterial-venous pH change (ΔpH_a-v_) relative to other vertebrates, may greatly enhance tissue oxygen delivery in teleosts (e.g., rainbow trout) during stress, beyond that in mammals (e.g., human). We generated oxygen equilibrium curves (OECs) at five different CO_2_ tensions for rainbow trout and determined that, when Hb-O_2_ saturation is 50% or greater, the change in oxygen partial pressure (ΔPO_2_) associated with ΔpH_a-v_ can exceed that of the mammalian Bohr effect by at least 3-fold, but as much as 21-fold. Using known ΔpH_a-v_ and assuming a constant arterial-venous PO_2_ difference (P_a-v_O_2_), Root effect Hbs can enhance O_2_ release to the tissues by 73.5% in trout; whereas, the Bohr effect alone is responsible for enhancing O_2_ release by only 1.3% in humans. Disequilibrium states are likely operational in teleosts *in vivo*, and therefore the ΔpH_a-v_, and thus enhancement of O_2_ delivery, could be even larger. Modeling with known P_a-v_O_2_ in fish during exercise and hypoxia indicates that O_2_ release from the Hb and therefore potentially tissue O_2_ delivery may double during exercise and triple during some levels of hypoxia. These characteristics may be central to performance of athletic fish species such as salmonids, but may indicate that general tissue oxygen delivery may have been the incipient function of Root effect Hbs in fish, a trait strongly associated with the adaptive radiation of teleosts.

## Introduction

Haemoglobin (Hb) is one of the most well studied proteins to date and is key to blood oxygen (O_2_) transport in nearly all vertebrates and some invertebrates, as it increases the total O_2_ that can be transported in the blood and optimizes tissue O_2_ delivery. The Bohr effect describes the reduction in Hb-O_2_ affinity when blood pH decreases and has been studied for over a century to understand how metabolic CO_2_ production can elevate the partial pressure of O_2_ (PO_2_) in the blood to enhance tissue O_2_ delivery [1–3]. The effect of pH on Hb-O_2_ can be graphically represented using an oxygen equilibrium curve (OEC), where Hb-O_2_ saturation decreases in a sigmoidal pattern depending on the PO_2_ of the system. With a given reduction in pH, as occurs between arterial and venous blood (ΔpH_a-v_) due to metabolic CO_2_ production, the OEC shifts to the right. Thus, at a given Hb-O_2_ saturation, such as P_50_ (PO_2_ at which Hb is 50% saturated), the shift in the OEC increases the PO_2_ at P_50_, which increases the driving force for tissue O_2_ delivery. The new P_50_ can be calculated using the following equation (Eq 1):

$$P_{50}=P_{50i}(10^{\Phi \Delta \text{pH}(\text{a}-\text{v})})$$ (1)

where P_50i_ refers to the initial P_50_ of the organism (e.g., under resting conditions) or system, Φ refers to the Bohr coefficient (Δlog P_50_/ΔpH), and ΔpH_a-v_ is the arterial-venous pH change. For example, a human with a P_50i_ of 27 mmHg [4], a Bohr coefficient of -0.35 [5], and ΔpH_a-v_ of -0.035 (a typical change that can occur *in vivo* [6]) will therefore exhibit a new P_50_ of 27.77 mmHg. While this is a ΔPO_2_ (ΔPO_2_ = P_50_—P_50i_) of less than 1 mmHg, it represents the increase in the driving force for tissue O_2_ delivery. End capillary PO_2_ values in mammals have been measured at 40 mmHg [7–10], and thus the ΔPO_2_ resulting from the Bohr effect would represent a modest benefit (~2%) to the driving force for tissue O_2_ delivery in a human.

Among vertebrates, teleosts possess Hbs whereby an acidosis not only greatly decreases Hb-O_2_ affinity, as in the Bohr effect, but also reduces the carrying capacity of Hb for O_2_, termed the Root effect [11–13]. The Root effect has long been discussed in terms of its role in enhancing O_2_ delivery to the eye and swimbladder of fishes. The eye and swimbladder are equipped with *retia*–dense capillary networks that can serve to localize and magnify an acidosis–that, in conjunction with the Root effect, greatly elevate arterial PO_2_ (P_a_O_2_) [14–16]. In the eye, the high P_a_O_2_ serves to overcome great diffusion distances to oxygenate the metabolically active, yet poorly vascularized retinal tissue [16–18]. At the swimbladder, a gas gland also aids in producing acid, and the resulting high P_a_O_2_ serves to inflate the swimbladder against large pressure gradients (>50 atm) associated with depth, permitting precise buoyancy regulation [14]. Teleosts that possess Root effect Hbs generally possess a large Bohr coefficient, much larger than occurs in air-breathing vertebrates with a Bohr effect alone. Consequently, it has been proposed that Root effect Hbs may also be in place to enhance O_2_ delivery to tissues in general, beyond the eye and swimbladder [2,19,20]. The focus of this study was to quantify the degree to which this may occur.

Indeed, the Root effect in teleosts is generally associated with a large Bohr coefficient, which is one step toward generating a large ΔPO_2_ during blood capillary transit; however, the other prerequisite is a large pH_a-v_, which under steady state conditions is thought not to occur [5]. This is because a Hb with a large Bohr coefficient also has a large Haldane coefficient, which minimizes or even prevents a pH_a-v_ difference due to binding of H^+^ upon Hb deoxygenation. However, recently it has been demonstrated that under some conditions teleosts may exhibit a large pH_a-v_ that may greatly exceed that of air-breathing vertebrates. If this is coupled with a large Bohr coefficient, O_2_ delivery could be greatly enhanced [21,22].

A large ΔpH_a-v_ may occur in teleosts during a generalized acidosis through the short-circuiting of red blood cell Na^+^/H^+^ exchange (NHE). During a generalized acidosis, most teleosts secure gill O_2_ uptake by protecting RBC pH via β-adrenergically stimulated NHE (βNHE), which upon activation, removes protons (H^+^) from the RBCs in exchange for Na^+^. However, Rummer and Brauner [23] determined that, *in vitro*, the βNHE and a general ‘housekeeping’ NHE could both be selectively short-circuited via plasma-accessible carbonic anhydrase (CA). This short-circuiting would create the large ΔpH_a-v_ needed to greatly enhance O_2_ delivery to select tissues. Short-circuiting was further validated *in vivo* in rainbow trout exposed to a mild acidosis (elevated water CO_2_) upon which muscle PO_2_ –determined using fast-responding fibre optic O_2_ sensors implanted directly into the fish’s muscle tissue–increased by 65% [21]. The increase in red muscle PO_2_ was completely abolished following arterial injection of a membrane-impermeant CA inhibitor, thus linking the increase in tissue PO_2_ to plasma-accessible CA [21]. Therefore, qualitative support exists for a large ΔpH_a-v_ and the associated effect on O_2_ delivery both *in vitro* and *in vivo*, which may only require a mild acidosis. However, the degree to which this may enhance O_2_ delivery during periods of severe environmental challenges, such as hypoxia or exercise, is not known.

At a given pH_a-v_ and Hb-O_2_ saturation (e.g., P_50_ or otherwise), a ΔPO_2_ (P_50_—P_50i_, where the respective level of saturation is used instead of 50%) can be calculated using Eq. 1 assuming that the Bohr coefficient is constant at the different Hb-O_2_ saturations. This is more or less the case in human blood, where under relevant *in vivo* conditions the shift in the OEC due to the Bohr effect is relatively constant between 20 and 80% Hb-O_2_ saturation [24]. However, Root effect Hbs exhibit a strongly non-linear release of Bohr protons with oxygenation [24], and consequently, the greatest Bohr shift exists between 60 and 100% Hb-O_2_ saturation [25–28]. Thus, a ΔPO_2_ in Root effect Hbs cannot simply be calculated as described above. Rather, ΔPO_2_ must be interpolated directly from OECs generated at constant pH values that span the *in vivo* range for that organism. Surprisingly, no such data set currently exists. Furthermore, there is tremendous variability in the literature regarding even the magnitude of the Bohr coefficient at P_50_, let alone at other Hb-O_2_ saturations, in rainbow trout (*Oncorhynchus mykiss*), despite it being one of the most comprehensively investigated teleost species to date. These inconsistencies may be in part due to differences in methodologies (Table A in S1 File).

We chose to thoroughly characterize the Root effect Hb system in rainbow trout, and our specific objectives were as follows: i) generate complete OECs in whole blood at constant pH values that span the *in vivo* range for rainbow trout; ii) calculate the ΔPO_2_ for a given proposed pH_a-v_ at set Hb-O_2_ saturations; iii) determine Bohr coefficients, Hill coefficients, and P_50_ values validated against other sampling and incubation techniques from our previous studies [29] to address the historical inconsistences that preclude many of these analyses otherwise; iv) compare this with the ΔPO_2_ calculated from models developed for a mammal (e.g., human) possessing a Bohr effect alone; v) estimate the increase in the O_2_ release from Hb and potential enhancement in tissue O_2_ delivery associated with a Root effect Hb that rainbow trout may experience during exercise or hypoxia, when oxygen delivery is most critical for performance. The latter may be particularly important in athletic species to speed up post-exercise recovery, following a predator-prey encounter, or during long, upstream migrations as exhibited in Pacific salmon [30], or to enhance O_2_ delivery in other species during exposure to environmental stress.

## Results

### Series 1: Influence of pH on the oxygen equilibrium curves of rainbow trout blood

**Fig 1:**
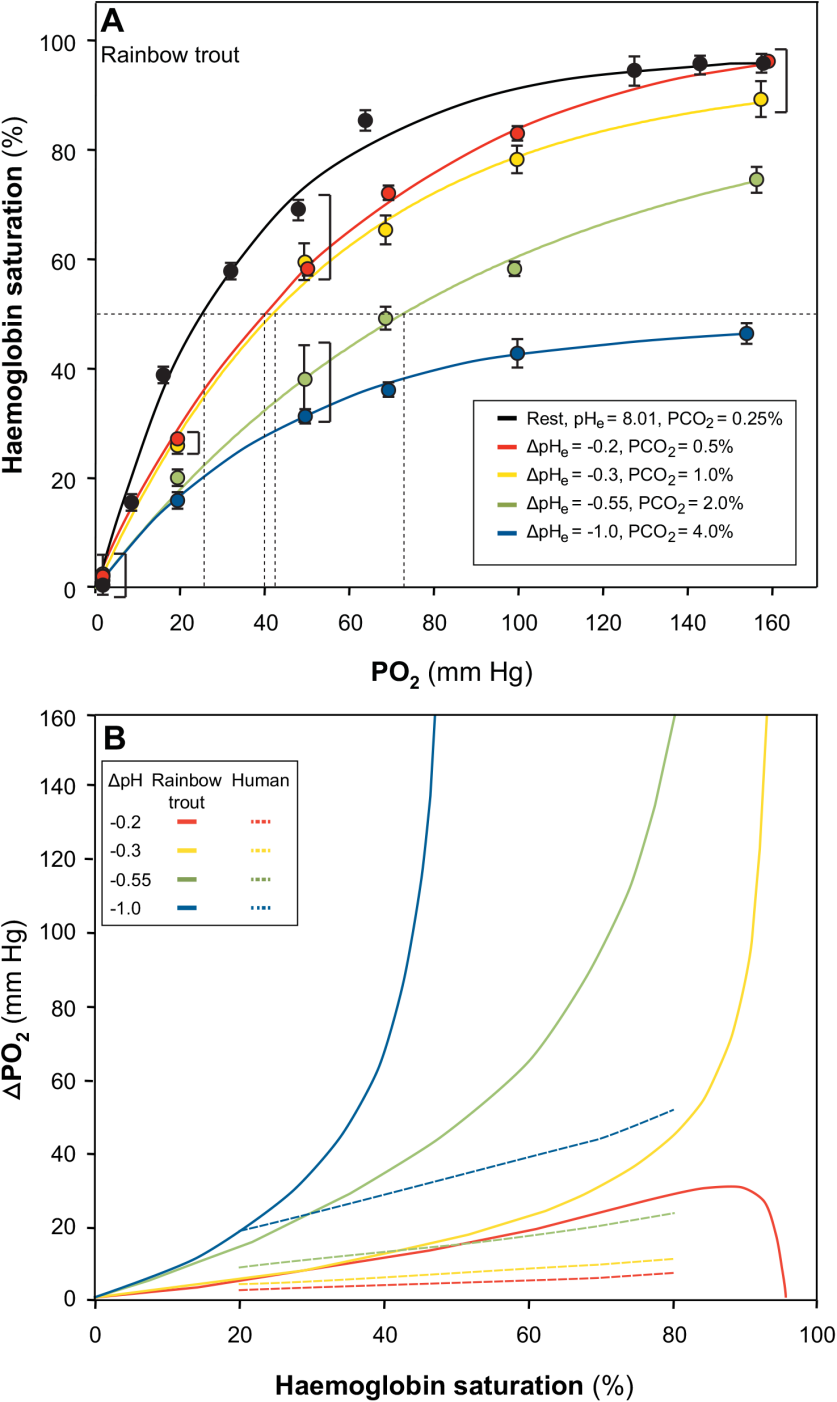
Oxygen equilibrium curves (OECs) generated at 0.25, 0.5, 1.0, 2.0, and 4.0% CO_2_ with stepwise decreases in PO_2_ (mmHg) from 160 to 0 mmHg (balance N_2_) for rainbow trout rinsed RBCs (panel A). The ΔpH_e_ relative to the 0.25% CO_2_ curve (black, pH_e_ = 8.01) is also noted for each treatment. Data are means ±S.E.M. Brackets indicate no statistically significant effect of CO_2_ at the respective PO_2_. Dashed lines extend from 50% Hb-O_2_ saturation to indicate the P_50_ for each OEC. In panel B, the magnitude of the right-shift of the OEC for a given pH change (ΔpH represented as different colours) is represented as ΔPO_2_ over each Hb-O_2_ saturation for a Bohr effect Hb system (human, dashed lines between 20 and 80% Hb-O_2_ saturation only) or a Root effect Hb system (rainbow trout, solid lines).

Blood was incubated at 0.25, 0.5, 1, 2, or 4% CO_2_ to simulate different pH_a-v_ over the entire range of Hb-O_2_ saturations. At each CO_2_ level and therefore pH, PO_2_ was also decreased stepwise, and mean Hb-O_2_ saturation was calculated at each step so that OECs could be generated. Mean Hb-O_2_ saturation significantly decreased with PO_2_ and with each increase in CO_2_ tension, resulting in the characteristic rightward (Bohr effect) and downward (Root effect) shifts in the OEC (Fig 1A). The exceptions were points generated from samples incubated at 0.25, 0.5, and 1% CO_2_ for a PO_2_ of 156 to 158 mmHg, which were not statistically different from each other. This pattern was evident again when samples were incubated at a PO_2_ of 47 to 49 mmHg. At air-saturated oxygen tensions (~160 mmHg) there was a reduction in Hb-O_2_ saturation from 95 and 96% at 0.25 and 0.5% CO_2_, to 89% at 1% CO_2_, 75% at 2% CO_2_, and 47% at 4% CO_2_ (Fig 1A), a reduction in carrying capacity that is characteristic of Root effect Hbs.

Blood samples were taken at each step of the incubation process for each curve generated. Both intracellular and extracellular pH (pH_i_ and pH_e_) were measured at each CO_2_ tension, and both decreased with higher levels of CO_2_ (P<0.001) (Table 1). Changes in pH_i_ were significantly and linearly correlated with changes in pH_e_ (pH_i_ = 2.747 + (0.574 * pH_e_), R^2^ = 0.930), similar to previous determinations with whole blood *in vitro* (pH_i_ = 2.708 + (0.595 * pH_e_), R^2^ = 0.950) [31]. Haematocrit (Hct) was measured in whole blood samples following each CO_2_ incubation and was found to slightly but significantly increase at 1, 2, and 4% CO_2_ relative to 0.25 and 0.5% CO_2_ incubations.

**Table 1:** Effects of carbon dioxide (% CO_2_) on haematological parameters, pH and oxygen transport-related variables for blood of rainbow trout.

**CO**_**2**_ **(%)**	0.25		0.5		1		2		4
**PCO**_**2**_ **(mm Hg)**	1.9		3.8		7.6		15.2		30.4
**[Hb] (mM)**	0.9 ±0.0^abc^		0.9 ±0.0^abc^		1.0 ±0.0^b^		1.0 ±0.0^b^		0.8 ±0.1^c^
**Hct (%)**	23.2 ±0.0^abd^		22.5 ±0.3^b^		25.3 ±0.8^c^		26.1 ±0.4^c^		24.5 ±0.2^cd^
**MCHC**	3.7 ±0.2^ab^		4.2 ±0.1^a^		4.1 ±0.1^a^		4.0 ±0.1^a^		3.1 ±0.4^b^
**pH**_**i**_	7.40 ±0.00^a^		7.24 ±0.05^b^		7.12 ±0.03^c^		6.96 ±0.05^d^		6.79 ±0.02^e^
**pH**_**e**_	8.01 ±0.00^a^		7.81 ±0.04^b^		7.69 ±0.02^c^		7.46 ±0.03^d^		6.96 ±0.01^e^
**P**_**50**_ **(mm Hg)**	24.8 ±0.3^a^		37.7 ±0.3^b^		40.6 ±2.2^b^		75.0 ±4.8^c^		>160.0
**n**_**H**_	1.3 ±0.1^a^		1.5 ±0.0^a^		1.4 ±0.0^a^		1.0 ±0.0^b^		0.6 ±0.1^c^
**Φ**		-0.91 ±0.18		-0.67 ±0.04		-0.87 ±0.06		-0.77 ±0.05	

Haemoglobin concentration [Hb], haematocrit (Hct), mean corpuscular haemoglobin concentration (MCHC), intracellular pH (pH_i_), extracellular or plasma pH (pH_e_), the Hill coefficient (n_H_), and the Bohr coefficient (Φ). All data were collected according to Series 1 protocols on rinsed RBCs. Letters that differ within rows indicate statistically significant effects of CO_2_.

Both 3-parameter logistic equations as well as Hill plots were used to calculate P_50_ values for each OEC, which were approximately 25, 38, 41, 75, and >160 mmHg (generated from 0.25 to 0.5, 1, 2, and 4% CO_2_ mixtures, respectively). Each were significantly different from one another (P<0.001) except for 0.5 and 1% CO_2_ samples (P = 0.05) (Table 1, S1 Fig, S1 File). Statistical analyses did not include the >160 mmHg P_50_ from samples; while the Hill plots resulted in a value of 162 mmHg, a nominal value could not be interpolated from the OECs. The Hill coefficient (n_H_) was also calculated for each curve, illustrating low cooperativity (1.34, 1.48, 1.37, 1.03, and 0.55 at 0.25, 0.5, 1, 2, and 4% CO_2_, respectively) that is characteristic of Root effect Hbs. The n_H_ using 2 and 4% CO_2_ were the only significantly different values (Table 1, S1 Fig, S1 File). Bohr coefficients for each CO_2_ incubation treatment were calculated at P_50_ and were -0.91, -0.67, -0.87, and -0.77 (at 0.25, 0.5, 1, 2, and 4% CO_2_, respectively) (Table 1).

### Series 2: Differences in ΔPO_2_ in a Bohr effect Hb system (human) and a Root effect Hb system (rainbow trout)

For the human model, the ΔPO_2_ was calculated as described above (Eq. 1), assuming a resting P_50_ of 27 mmHg [4], Φ of -0.35 [5] and assuming Φ was constant at all pH values between 20 and 80% Hb-O_2_ saturation, as this is where the shift in the OEC due to the Bohr effect is relatively constant [24]. The PO_2_ values at P_20_, P_30_, P_40_, P_60_, P_70_, and P_80_ were extrapolated from a Hill plot generated using n_H_ = 2.8 and P_50_ = 27 mmHg. A ΔpH of -0.2, -0.3, -0.55 and -1.0 pH units were chosen to be consistent with those values determined for rainbow trout. While the latter two values far exceed what might be seen *in vivo*, they serve to illustrate the dramatic differences between the two model systems investigated. It was assumed that Hb always reached 100% saturation at atmospheric O_2_ tensions for the human model because they do not possess Root effect Hbs. Therefore, the model was restricted to Hb-O_2_ saturations between 20 and 80%, as a ΔPO_2_ would not be expected at 0 and 100% SO_2_. Rainbow trout blood ΔPO_2_ values were obtained by direct interpolation from the OECs generated in Series 1 (Fig 1A). The SO_2_ values from 0 to100% for a ΔpH_e_ of 0.1 (by comparing the 0.25 and 0.5% CO_2_ OECs), ΔpH_e_ of 0.2, (by comparing the 0.25 and 1% OECs), ΔpH_e_ of 0.5 (by comparing the 0.25 and 2% CO_2_ OECs), and ΔpH_e_ of 1.0 (by comparing 0.25 and 4% CO_2_ OECs). The ΔPO_2_ depending on Hb-O_2_ saturation for human and rainbow trout determined at the same ΔpH values are shown superimposed in Fig 1.

For human blood between 20 and 80% Hb-O_2_ saturation, the ΔPO_2_ values for a ΔpH of -0.2, -0.3, -0.55, and –1.0 were relatively constant (because the Bohr coefficient was assumed constant over this range) and were 4.7, 7.4, 15.1, and 33.4 mmHg, respectively at P_50_. For rainbow trout blood, the ΔPO_2_ values directly interpolated from the OECs for ΔpH values of -0.2, -0.3, -0.55, and –1.0 ranged from 14.6 to 295.1 mmHg at 50% Hb-O_2_ saturation. Thus, at a given ΔpH, the ΔPO_2_ depended greatly on the Hb-O_2_ saturation. The ΔPO_2_ for each ΔpH was on average 2.5-times greater in rainbow trout blood than human blood at 40% Hb-O_2_, and this difference increased at higher saturations. For Hb-O_2_ saturations up to 80% and a ΔpH close to what might be expected *in vivo* in trout (0.2 pH units), the ΔPO_2_ for rainbow trout was, at minimum, 4-fold that of the human ΔPO_2_ and over 21-times greater at greater ΔpH values (Fig 1B).

### Series 3: Modeling changes in O_2_ release from Hb in a Bohr effect system in comparison to a Root effect system.

**Fig 2:**
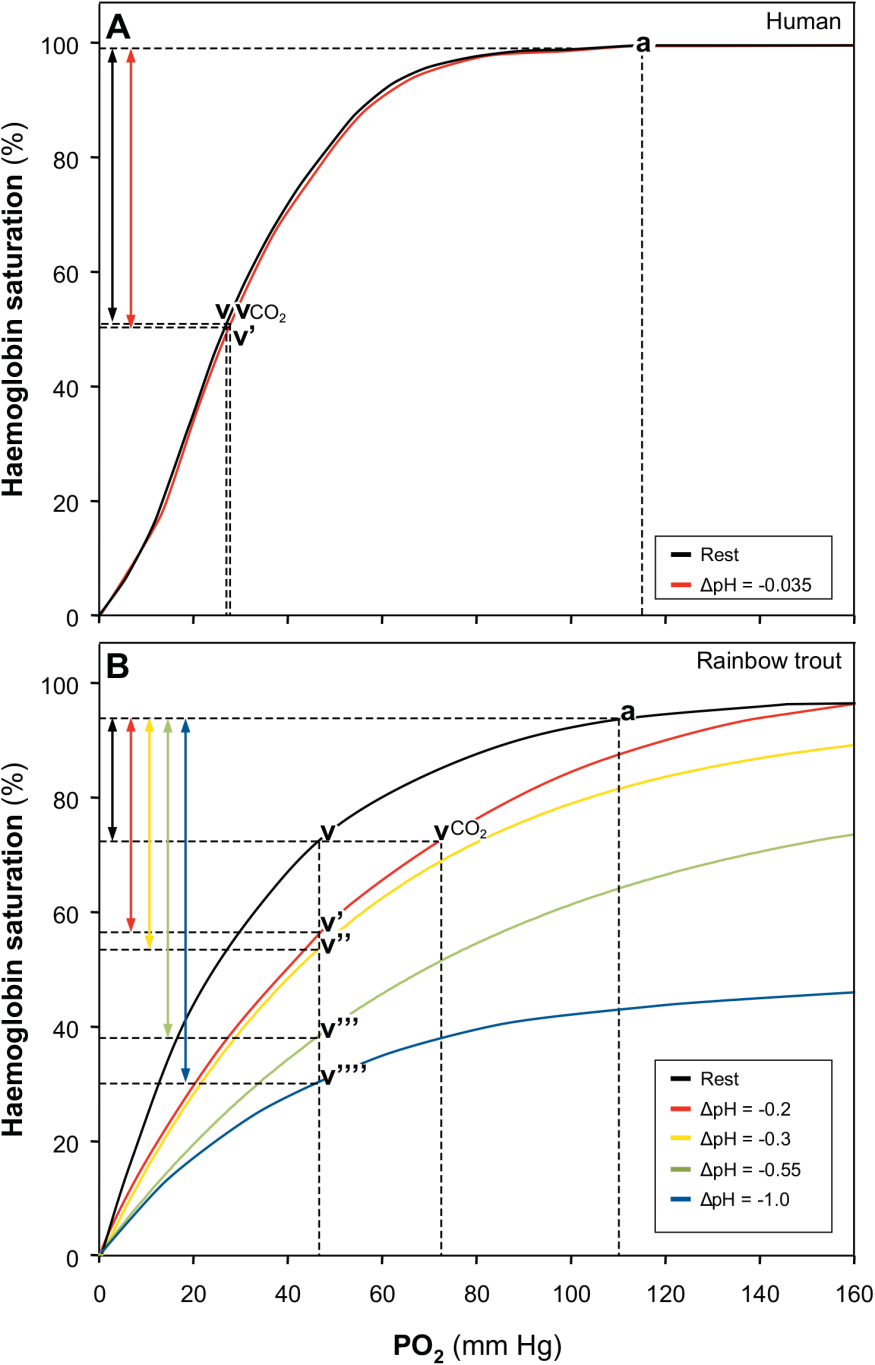
The degree to which O_2_ release from Hb is enhanced for a given ΔpH for a Bohr effect Hb system (human, panel A) and a Root effect Hb system (rainbow trout, panel B). The increase in O_2_ release with respective right-shifts in the OECs is represented by vertical double arrows here and as a percent increase over what is possible without the right shift in the text (see above section “Series 2: Differences in ΔPO_2_ *…*” for details).

The OECs generated for human and trout blood were used to model O_2_ release from Hb and tissue O_2_ delivery. For the human model, a P_a_O_2_ of 115 mmHg (labeled “a” on Fig 2A), a P_v_O_2_ of 27 mmHg (labeled “v” on Fig 2A), and a physiologically relevant ∆pH_a-v_ of 0.035 were used. All above values correspond to values obtained from previous studies [6,32–35]. The corresponding right-shifted OEC was plotted assuming a constant Bohr coefficient, Φ = -0.35, between 20 and 80% Hb-O_2_ saturation [4]. For the rainbow trout model, a P_a_O_2_ of 110 mmHg was used (labeled “a” on Fig 2B), and P_v_O_2_ (labeled “v” on Fig 2B) was estimated from muscle O_2_ values of 45–47 mmHg, both of which correspond to values obtained *in vivo* [21]. The OEC curves generated from the current study were used to simulate the various ∆pH_a-v_ for the model and also to accommodate the non-linear Bohr shift known to occur at the different Hb-O_2_ saturations in teleosts.

Oxygen extraction over a given P_a-v_O_2_ and without a right-shift in the OEC (“a” to “v”) is represented by a black vertical double arrow for both human and trout models (Fig 2). The increase in O_2_ released from Hb that is associated with a given ∆pH_a-v_ and resulting right shift in the OEC can then be estimated by tracing “v” horizontally to the first right-shifted OEC to the point labeled “vCO_2_” and then moving down this new OEC to the P_v_O_2_ estimate corresponding with v’ on the model. For the human, the increase in O_2_ released from Hb as a result of the right-shift in the OEC (a to v’) would be a 1.3% increase from what would occur from “a to v” (i.e., without a right-shift, compare black and red vertical arrows; Fig 2A). For rainbow trout (Fig 2B), the increased O_2_ extraction as a result of the right-shift in the OEC (a to v’) would be 73.5% (compare black and red vertical arrows; Fig 2B). Thus, under these conditions, there is over a 50-fold increase in the additional O_2_ released from the Hb associated with the right shift in the OEC in trout relative to the human model. Under a more severe acidosis, the OEC shifts further to the right corresponding to v”, v”‘, or v”“and a respective 88, 160, or 197% increase or a doubling to tripling of O_2_ released from Hb (compare black to yellow, green, or blue vertical arrows; Fig 2B) and therefore potentially tissue O_2_ delivery.

**Fig 3:**
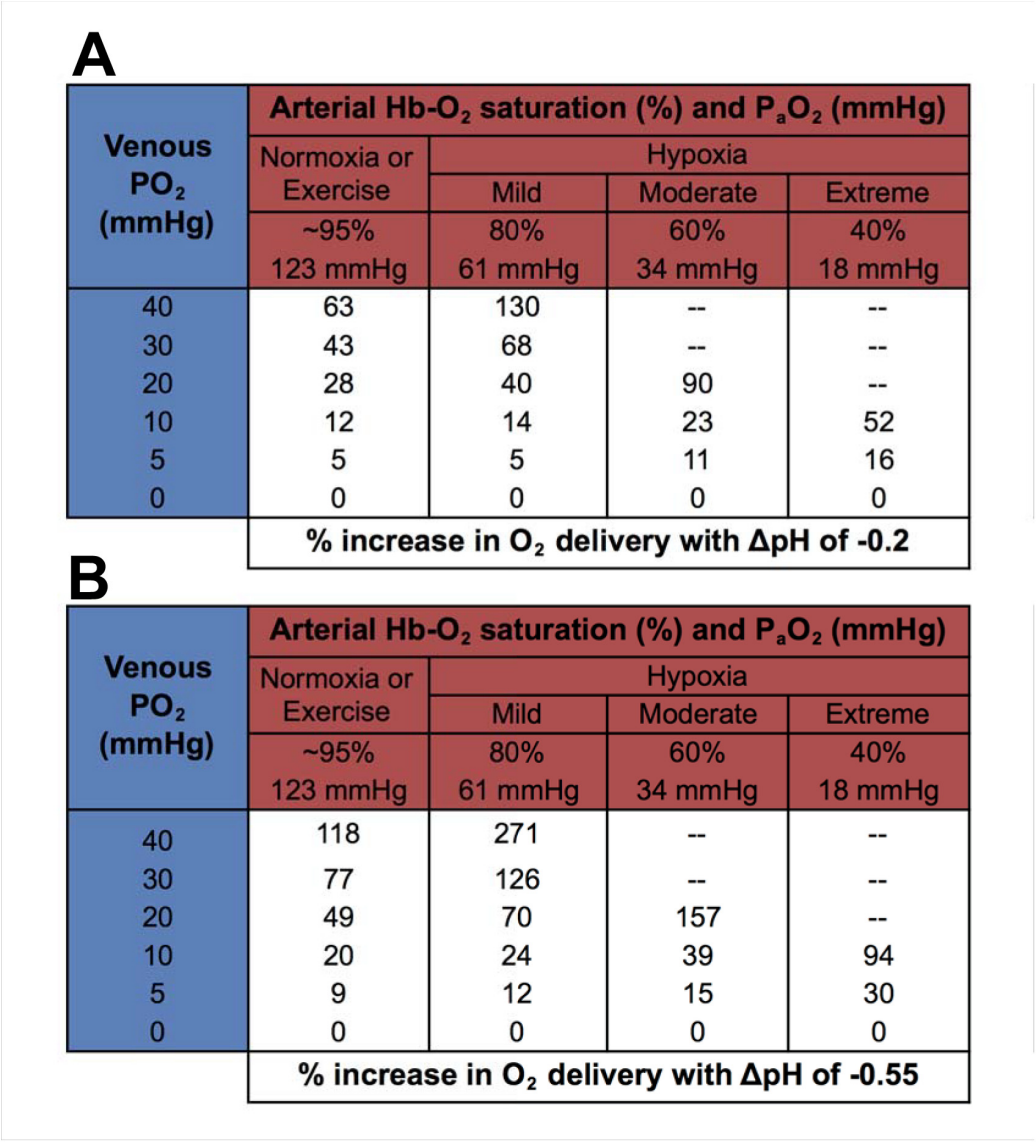
Model estimates for enhanced O_2_ release from Hb (as a %) in rainbow trout under normoxic conditions, various levels of sustained exercise, and various levels of hypoxia at two different simulated ∆pH_a-v_ (panels A and B) compared to when there is no right shift in the oxygen equilibrium curve (OEC). The P_a_O_2_ values corresponding to arterial Hb-O_2_ saturations (indicated by red cells) were derived from the resting curve of Fig 1A. Calculated values at each combination of arterial Hb-O_2_ saturation (red cells) and venous PO_2_ (P_v_O_2_) values (blue cells) represent the % increase in O_2_ delivery (white cells) over what is possible without a right-shift in the OEC. Curves for ∆pH = -0.2 (panel A) and ∆pH = -0.55 (panel B) were used for this model. For scenarios where the P_a-v_O_2_ difference was less than 5 mmHg (deemed unrealistic), no data are shown (—).

An additional model was generated for rainbow trout to predict the degree to which O_2_ release from Hb could be enhanced under normoxic conditions, at various levels of sustained exercise, or during exposure to hypoxia (Fig 3). We used P_a_O_2_ values corresponding to ~95% Hb-O_2_ saturation as well as various levels of hypoxia (e.g., 80, 60, and 40% Hb-O_2_ saturation) and a range of P_v_O_2_ levels (40, 30, 20, 10, 5, and 0 mmHg). Both ∆pH = -0.2 and ∆pH = -0.55 OEC curves were used to represent two potential ∆pH_a-v_ scenarios. The first could be experienced *in vivo* at the tissues in the presence of a mild acidosis [21,23], and the second would represent a more severe acidosis–both cases representing involvement of selective short-circuiting of RBC NHE via plasma-accessible CA. The % increase in O_2_ release with Root effect Hbs, depending on the scenario, is most often at least 40% or greater and in five scenarios exceeds a 100% increase in O_2_ release (Fig 3). Furthermore, the increase in O_2_ release from Hb is almost doubled when the ∆pH is -0.55 when compared to ∆pH of -0.2 (Fig 3).

## Discussion

The present study quantifies the potential benefit to tissue O_2_ delivery associated with a ∆pH_a-v_ in the presence of Root effect Hbs (rainbow trout) compared to that for a mammalian Bohr effect Hb system (human). Overall, it was determined that the ΔPO_2_ occurring in rainbow trout blood at 40% Hb-O_2_ saturation was 2.5-fold greater than that of a system with only a Bohr effect, using the same ∆pH_a-v_ (Fig 1B). At Hb-O_2_ saturations greater than 50%, the difference was even more pronounced, 21-times that of a system possessing only a Bohr effect (Fig 1B). Upon modeling O_2_ release from Hb, it was determined that a ΔpH_a-v_ of 0.2 in rainbow trout blood could increase O_2_ extraction by 73.5% at constant P_v_O_2_ (Fig 2B). Based on ΔPO_2_ values calculated from OECs associated with larger changes in pH, it follows that the enhancement in O_2_ release from Hb in rainbow trout could be even greater. In the human, however, a physiologically relevant pH change under otherwise similar assumptions may only increase O_2_ release from Hb by 1.3% (Fig 2A). This is the first time this difference has been quantified to this extent, despite qualitative predictions [19,26,36]. It follows that, if a relatively large ΔpH_a-v_ can be generated in the general circulation of fish, which has been recently demonstrated *in vitro* and *in vivo* in rainbow trout [21,23], Root effect Hbs could be responsible for greatly enhancing general O_2_ delivery. Modeling with known P_a-v_O_2_ in fish during exercise and hypoxia indicates that O_2_ release from Hb and thus tissue O_2_ delivery may double during exercise and even triple during some levels of hypoxia.

One of the assumptions associated with the model we present is that fish exhibit larger pH_a-v_ differences than air-breathing vertebrates. To be conservative, many of the estimates presented here are based upon measured pH_a-v_ differences in fish; although, recent data suggest that the pH_a-v_ difference realized at the tissues under periods of stress are likely to be even larger [21,22]. This is due to the presence and then elimination of disequilibrium states [22], which are almost impossible to measure directly. It is also important to note that intracellular pH (pH_i_) cannot yet be reliably measured in real-time at the tissue/cellular level *in vivo*, and disequilibrium states in the blood preclude accurate representations of pH_i_ once blood is collected and RBCs are lysed for measurement. So, even though many past studies have drawn arterial and venous blood samples for pH measurements, the values determined are equilibrium values that may differ greatly from pH values realized at the tissues. Currently, the fastest and most sensitive way to infer changes in RBC pH_i_ is via changes in PO_2_ that we know occur as a result of a decrease in pH_i_, and indeed, the technology is already available for real-time measurements of PO_2_ at the tissues.

The magnitude of the ΔpH_a-v_ that is possible in teleosts has recently been described and elaborated upon by Randall et al., and the sequence of events contrast the model for air-breathing vertebrates in several ways [22]. In teleosts, there is a loss of CA at the respiratory surface and in the venous circulation, an uncoupling of RBC and plasma pH, and a decrease in the role of Hb as a buffer. This collectively results in nearly all plasma bicarbonate dehydration occurring inside the RBC, which results in disequilibrium states that elevate arterial and venous bicarbonate levels [22]. With the presence of plasma-accessible CA near some tissues, disequilibrium states can be eliminated to form CO_2_ that re-acidifies the RBCs. All of this can be greatly magnified during acidotic stress and has been supported both *in vitro* [23] and *in vivo* [21]. In rainbow trout exposed to a mild acidosis (0.2 pH unit reduction in blood pH), for example, red muscle PO_2_ increased to an extent nearly identical to the right shift in the OECs derived from this study with the same pH change (0.2 pH unit). The ΔPO_2_ was abolished when plasma accessible CA in the red muscle was selectively inhibited [21]. These findings suggest that–at least during a mild acidosis–an entire acid load can be transferred from the plasma into the RBC, resulting in the equivalent of a ΔpH_a-v_ of 0.2 pH units *in situ*. This is a much larger difference than would be inferred from arterial and venous blood drawn and then measured at equilibrium, which represents most of the literature values. Whether the same applies under more severe acidoses *in vivo*, as it does *in vitro* [23], needs to be investigated. However, the predictions generated here can now be tested experimentally. Clearly there is a tremendous potential for enhanced O_2_ release from the Hb associated with a large ∆pH_a-v_ and potentially tissue O_2_ delivery.

### Past information on haemoglobin-oxygen relationships in rainbow trout

Rainbow trout is arguably one of the most universally investigated teleost species with respect to O_2_ transport and respiratory physiology. The absence of a comprehensive data set sufficient to model ΔPO_2_ over a range of ΔpH (Table A in S1 File) was therefore surprising. An extensive review of the available literature reveals that reported O_2_ transport-related variables in rainbow trout blood are highly variable. For example, great variability in Bohr coefficients are observed ranging from –0.15 to –1.97 (Table A in S1 File, S2 Fig, S1 File) and P_50_ values for control or resting animals as low as 11 but as high as 40 mmHg (Table A in S1 File). This variability is likely due to the range of animal holding conditions, sampling techniques, blood preparation and protocols (Table A in S1 File), but it is unknown how these differences may affect the values those authors obtained. Consistent OECs and calculated Bohr and Hill coefficients and P_50_ values were generated using two different blood sampling protocols (rinsed RBCs or whole blood drawn from an indwelling dorsal aortic cannula), and two methods of equilibrating blood with gases (tonometry and the Tucker method vs. a modified version of the microdiffusion chamber and spectrophotometer, i.e. the P_wee_50) [29] to further validate the data used for the models in the present study.

### Modeling increased O_2_ release from Hb and tissue O_2_ delivery with a Bohr effect Hb (human) and a Root effect Hb (rainbow trout)

Christiansen and colleagues calculated a maximal P_50_ shift (ΔP_50_) in human blood associated with the Bohr effect *in vivo* of 3 mmHg, and so the predicted benefit to O_2_ delivery associated with the Bohr effect alone is quite modest. In modeling ΔPO_2_ associated with a pH change in human blood, a Bohr coefficient of –0.35 was assumed, a value deemed optimal for O_2_ delivery [5]. This value also falls within the middle of the range of fixed-acid and CO_2_ Bohr coefficients calculated from whole blood of healthy humans [37]. It was also assumed that the Bohr coefficient was constant between 20 and 80% SO_2_ [24]. A P_50_ value of 27 mmHg was chosen, which is midway between resting values of 24 and 29 mmHg, which have previously been determined at a pH of 7.4 [4,38–41]. The ΔpH values chosen were consistent with those determined in Series 1 for rainbow trout OECs to allow the two systems to be compared in terms of the contribution of a ΔpH_a-v_ to enhancing O_2_ release from Hb. Our models may actually underestimate the difference between the two systems, however, because pH is a log scale, and starting pH values are lower in humans (7.4) than in rainbow trout (8.0). As such, more H^+^ would be added to the human system, which has a lower H^+^ sensitivity, than the trout system, despite using the same ΔpH. Therefore, if the same number of H^+^ were added to the trout system, the result may be even greater than we predict here. The measured ΔpH_a-v_ in humans, 0.035 pH units, is quite small relative to even the lowest ΔpH value of 0.2 units used in this study, which between 20 and 80% Hb-O_2_ saturation, results in a ΔPO_2_ of less than 8 mmHg. A ΔpH of 0.035 would result in a ΔPO_2_ of even less than 1 mmHg, which would increase O_2_ release from Hb by 1.3%. However, it should also be noted that mammals can have nearly double the blood Hb concentrations of teleosts, which must be taken into account in determining the increase in tissue O_2_ delivery that may be realized with this modest Bohr effect. On the other hand, teleosts may be able to exploit this phenomenon that we describe here to enhance tissue O_2_ delivery while maintaining lower blood Hb concentrations.

In comparison, O_2_ release associated with a ΔpH_a-v_ in teleost blood with Root effect Hbs is much greater than that in an air-breathing vertebrate with a Bohr effect Hb only, provided a sufficient pH change in the blood is observed. This varies with blood Hb-O_2_ saturation for a number of reasons. First, the Root effect results in incomplete Hb-O_2_ saturation at low pH, despite atmospheric PO_2_ levels [11–13]. This was evident in the OECs generated for rainbow trout, as larger pH changes resulted in curves that approached apparent upper asymptotic maximum Hb-O_2_ saturations that were distinctly and significantly lower than 100% (Fig 1A). This translated to very high ΔPO_2_ values upon interpolation (Fig 1B). Second, Root effect Hbs typically exhibit large Bohr coefficients [19,26,36]. As a result, the ΔPO_2_ in rainbow trout blood exceeds that of human blood at all comparable ΔpH values between 20 and 80% Hb-O_2_ saturation (Fig 1B). A 0.2 ΔpH_a-v_ difference in human blood is unlikely, but a difference of this magnitude has been measured in rainbow trout during exercise [42,43]. This is also a value that can be realized at the level of the RBC when disequilibrium states are eliminated in the presence of plasma-accessible CA at the tissues, as described above. Even at a ΔpH_a-v_ of 0.035, the ΔPO_2_ in rainbow trout blood still exceeds that of the human blood by as much as 3-fold (data not shown). Third, Root effect Hbs exhibit a non-linear release of Bohr protons with Hb-O_2_ saturation, and therefore the magnitude of the Bohr shift varies with Hb-O_2_ saturations [26,44]. This is apparent in Fig 1B, where ΔPO_2_ for a given ΔpH_a-v_ is much greater in rainbow trout blood than human blood above P_50_, but this difference decreases below P_50_ (Fig 1B).

To date, only two studies have monitored real-time muscle PO_2_ in a teleost, and both of these studies provided evidence that tissue O_2_ delivery may be enhanced relative to other vertebrates [20,21]. Whether during normoxia, mild hypoxia, mild hypercarbia, or sustained or exhaustive exercise, red muscle PO_2_ in rainbow trout (between 45 and 60 mmHg; [20,21]) is consistently and substantially higher than in mammals (25–35 mmHg [7–10]), and this difference has been proposed to be due to the presence of Root effect Hbs. Furthermore, the fact that muscle PO_2_ still remains elevated during stress (hypoxia, hypercarbia, or exercise [20,21]) and above P_v_O_2_ (approximately 20 mmHg; [45]), despite decreases in P_a_O_2_, also suggests the importance of Root effect Hbs to enhancing O_2_ delivery. Here, the comparative model for human and trout with relevant ΔpH_a-v_ demonstrated at least a 50-fold difference in the impact that Root effect Hbs have on enhancing O_2_ release from Hb when compared to a system with only a Bohr effect.

The potential for enhanced O_2_ delivery in rainbow trout was calculated using arterial Hb-O_2_ saturation, P_a_O_2_, and P_v_O_2_ values that represent normoxia, sustained exercise, and two levels of hypoxia associated with the exploitation of two different ΔpH_a-v_ (Fig 3). The associated increase in O_2_ release from Hb over a given P_a-v_O_2_ difference is remarkable, resulting in an increase of at least 40% or greater and in five scenarios exceeds a 100% increase (Fig 3). Assuming that, under these conditions, all other aspects of the oxygen cascade remain constant, and in particular there is no change in tissue metabolism or perfusion, this increase in O_2_ release would be directly proportional to the increase in tissue O_2_ delivery. Ultimately, this model serves to illustrate the remarkable potential for enhanced O_2_ delivery under various scenarios in a system unique to teleosts. Enhanced O_2_ delivery may be particularly important during exposure to environmental stress or prolonged exercise–for example–during the long, upstream migrations that the Pacific salmon undertake [30] or even to speed up post-exercise recovery, such as following a predator-prey interaction.

## Conclusions

Quantitative results confirmed theoretical predictions that–for a given pH change–Root effect Hbs in rainbow trout convey an enormous benefit to blood O_2_ release and thus delivery when compared with human blood having a Bohr effect alone. Teleost fish evolved an extraordinary O_2_ delivery system associated with the extremely pH-sensitive Root effect Hbs. This system has been understood for decades as key to O_2_ delivery to the eye and swimbladder, which may have been important factors responsible for the extensive adaptive radiation of teleosts. Provided here is empirical evidence to suggest that Root effect Hbs, in conjunction with a mechanism to increase pH_a-v_ (via the presence of plasma accessible CA in the tissues and absence at the gills), can also enhance *general* O_2_ delivery, which is consistent with recent *in vitro* and *in vivo* studies [20,21,23]. It may be that Root effect Hbs, which evolved prior to the appearance of the anatomical structures (*retia*) at the eye and swimbladder typically associated with this exceptional O_2_ delivery system, were initially selected for enhancing *general* O_2_ delivery through the associated large Bohr coefficient and large pH_a-v_ difference. Studies on a model species, such as the rainbow trout, that is neither basal nor the most derived of the teleosts and exhibits a moderate level of activity and tolerance to environmental conditions provides a foundation on which to build further studies to understand how evolutionary history, activity, and habitat may play a role in the functional significance of this system.

## Materials and Methods

### Series 1: Influence of pH on the oxygen equilibrium curves of rainbow trout blood

#### 1.1 Experimental animals and holding conditions

Rainbow trout, (*O*. *mykiss*, 300–600g wet body mass), were obtained from Spring Valley Trout Farm (Langley, British Columbia, Canada) and maintained at the University of British Columbia (UBC) Aquatic Facilities. Fish were held under a natural photoperiod at densities no greater than 10kg/m^3^ [46] in 4,000-l tanks supplied with flow-through 10°C Vancouver dechlorinated municipal tap water. Fish were fed every other day to satiation using commercial trout pellets (Skretting, Orient 4–0). Experiments were completed within the spring months over two separate years. All procedures complied with the guidelines approved by the Canadian Council on Animal Care and were approved by UBC’s animal ethics care and use committee (UBC protocol approval # A07-0080).

#### 1.2 Caudal puncture sampling protocol

Fish were quickly removed from holding tanks and placed into a 20 l bucket of clean, well-aerated water containing benzocaine (0.2 mM final concentration, p-aminobenzoate, Sigma-Aldrich cat. no. E1501; St. Louis, MO, USA) to anaesthetize fish. Blood was drawn from the caudal vein and collected in heparinized syringes, and RBCs were rinsed twice and resuspended in ice-cold Cortland's saline [47] according to Caldwell et al. [48]. Haematocrit (Hct) of the rinsed RBCs was measured in duplicate by centrifuging 60μl whole blood in heparinized micro-capillary tubes for 3 min at 17,000 g and was standardized to 25% by removing either saline or RBCs. Blood was stored at 4°C overnight until experiments commenced, ensuring that any catecholamines present within the sample had degraded [49].

#### 1.3 Oxygen equilibrium curves derived from tonometry

The Hct of rinsed RBCs stored at 4°C overnight was readjusted to 25% as needed. Then, 4 ml was added to each of four Eschweiler tonometers, which were incubated at 12°C, and equilibrated for one hour with a humidified gas mixture to one of the following CO_2_ proportions: 0.25, 0.5, 1, 2, or 4% balanced with air (21% O_2_). To generate an OEC at each of the above % CO_2_ values (n = 8), each tonometer was subjected to a step-wise decrease in O_2_ (21, 20, 19, 13, 9, 6.5, 4, 2.5, 1.5, or 0%) balanced with N_2_ using a DIGAMIX Wösthoff gas-mixing pump (DIGAMIX 275 6KM 422 Wösthoff, Bochum, Germany). Following a 20-min incubation period at each O_2_ tension, two 25 μl aliquots of rinsed RBCs were withdrawn into a pre-gassed Eppendorf™ tube or Hamilton™ syringe for measurement of total O_2_ content (TO_2_), and up to a further 500μl was withdrawn to measure haemoglobin concentration ([Hb]), Hct, extracellular pH (pH_e_), and intracellular pH (pH_i_). TO_2_ was measured according to Tucker [50], [Hb] (mM per tetramer) was measured after adding rinsed RBCs to Drabkin’s solution (Sigma-Aldrich cat. no. D5941; St. Louis, MO, USA), measuring absorbance at 540 nm, and applying a millimolar extinction coefficient of 11. The freeze-thaw technique [51] was used to measure pH_i_, where both pH_e_ and pH_i_ were measured using a BMS 3 Mk2 Blood Microsystem in conjunction with a PHM 84 meter (Radiometer, Copenhagen). A larger blood volume (500μl) was drawn when pH was measured, which was done at the highest, middle, and lowest O_2_ incubation tensions. All assays were performed in duplicate.

#### 1.4 Calculations and statistical analyses

Data are presented as means ±S.E.M. Mean corpuscular haemoglobin concentration (MCHC) was calculated as Hb/(Hct/100). Haemoglobin % saturation (SO_2_) was calculated by dividing TO_2_ (after subtracting physically dissolved O_2_ according to [52]) by the theoretical maximum carrying capacity of the rinsed RBCs based upon the tetrameric Hb concentration obtained spectrophotometrically according to [50]. The SO_2_ values were plotted as a function of incubation PO_2_ (mmHg) for each % CO_2_ (0.25, 0.5, 1.0, 2.0, and 4.0%), and a curve was fit to the data using the Dynamic Fit Wizard function in SigmaPlot for Windows 13.0 (Systat Software Inc.) to generate the OECs. The P_50_ and Hill coefficients (n_H_) were calculated from Hill plots. Bohr coefficients (Φ) were calculated as ∆logP_50_/∆pH_e_ for pH values corresponding to each CO_2_ incubation condition relative to 0.25% CO_2_. Statistical differences among CO_2_ treatments were detected via ANOVA and, when necessary, a post-hoc Holm-Sidak multiple comparisons test. All statistical analyses were conducted using SigmaPlot for Windows 13.0 (Systat Software, Inc.), using α < 0.05 to determine statistical significance.

### Series 2: Differences in ΔPO_2_ in a Bohr effect Hb system (human) and a Root effect Hb system (rainbow trout)

#### 2.1 Human model with a Bohr effect Hb system

The ΔPO_2_ was calculated (using Eq. 1), assuming a P_50_ of 27 mmHg [4], Φ of -0.35 [5], and assuming Φ was constant at all pH values between 20 and 80% SO_2_. A ΔpH of -0.2, -0.3, -0.55 and -1.0 pH units were chosen to be consistent with those values determined for rainbow trout below, and while the latter two values far exceed what might be seen *in vivo*, they serve to illustrate the dramatic differences between the two model systems investigated. It was assumed that Hb always reached 100% saturation at atmospheric O_2_ tensions. Therefore, the ΔPO_2_ at 0 and 100% SO_2_ always equalled zero.

#### 2.2 Rainbow trout model with a Root effect Hb system

Rainbow trout blood ΔPO_2_ values were obtained by direct interpolation from the OECs generated in Series 1. The SO_2_ values from 0 to100% for a ΔpH_e_ of 0.1 (by comparing the 0.25 and 0.5% CO_2_ OECs), ΔpH_e_ of 0.2, (by comparing the 0.25 and 1% OECs), ΔpH_e_ of 0.5 (by comparing the 0.25 and 2% CO_2_ OECs), and ΔpH_e_ of 1.0 (by comparing 0.25 and 4% CO_2_ OECs). The same four pH shifts (ΔpH) simulated in rainbow trout blood were used for the human blood calculations as described above.

### Series 3: Modeling changes in O_2_ release from Hb in a Bohr effect system in comparison to a Root effect system.

The OECs generated at different CO_2_ levels from this study were used to calculate the increased O_2_ release from Hb associated with a given ∆pH_a-v_ and a constant ∆P_a-v_O_2_. Then, O_2_ release can be used to estimate enhanced tissue O_2_ delivery associated with the respective ∆pH_a-v_, assuming that all other aspects of the O_2_ transport cascade were unaffected (e.g., including tissue metabolic rate and blood flow).

For the human model, a P_a_O_2_ of 115 mmHg, P_v_O_2_ of 27 mmHg, and a physiologically relevant ∆pH_a-v_ of 0.035 were used because they correspond to values obtained from previous studies [6,32–35]. The corresponding right-shifted OEC was plotted assuming a constant Φ = -0.35 between 20 and 80% Hb-O_2_ saturation [4]. For the rainbow trout model, a P_a_O_2_ of 110 mmHg was used, which corresponds to values obtained *in vivo* in rainbow trout [21]. P_v_O_2_ was assumed constant and estimated from RMPO_2_ values (45–47 mmHg, [21]), which closely resemble P_v_O_2_. The OEC curves generated from the current study were used to simulate the various ∆pH_a-v_ for the model and because PO_2_ values at each Hb-O_2_ could not be calculated using Eq. 1 because of the non-linear Bohr effect precluding a constant Φ at different Hb-O_2_ saturations.

An additional model was generated for rainbow trout to predict the degree to which tissue O_2_ delivery could be enhanced under normoxic conditions, hypoxic conditions, or at various levels of sustained exercise. We used P_a_O_2_ values corresponding to ~95% Hb-O_2_ saturation as well as various levels of hypoxia (e.g. 80, 60, and 40% Hb-O_2_ saturation) and a range of P_v_O_2_ levels (40, 30, 20, 10, 5, and 0 mmHg). Both ∆pH = -0.2 and ∆pH = -0.55 OEC curves were used to represent two potential ∆pH_a-v_ that could be experienced *in vivo* at the tissues [21,23].

## Supporting Information

S1 Fig (EPS)

S2 Fig (EPS)

S1 File Includes Table A. (DOCX)
