# Supplementary material for: Root Effect Haemoglobins in Fish May Greatly Enhance General Oxygen Delivery Relative to Other Vertebrates
Source: PLoS One. 2015 Oct 5;10(10):e0139477. doi: 10.1371/journal.pone.0139477 (PMC4593521; doi:10.1371/journal.pone.0139477)
Supplement: S1 File — (DOCX) [file pone.0139477.s003.docx]

# Supporting Information

**Metadata are available here:** doi:10.5061/dryad.d0325

Files included:

All trout OEC curve data and fits

Human oxygen equilibrium curves from the literature

All trout delta PO_2_ values

All human delta PO_2_ values

**Table A. Oxygen transport-related variables from previous data published on rainbow trout [41,53-68].** **
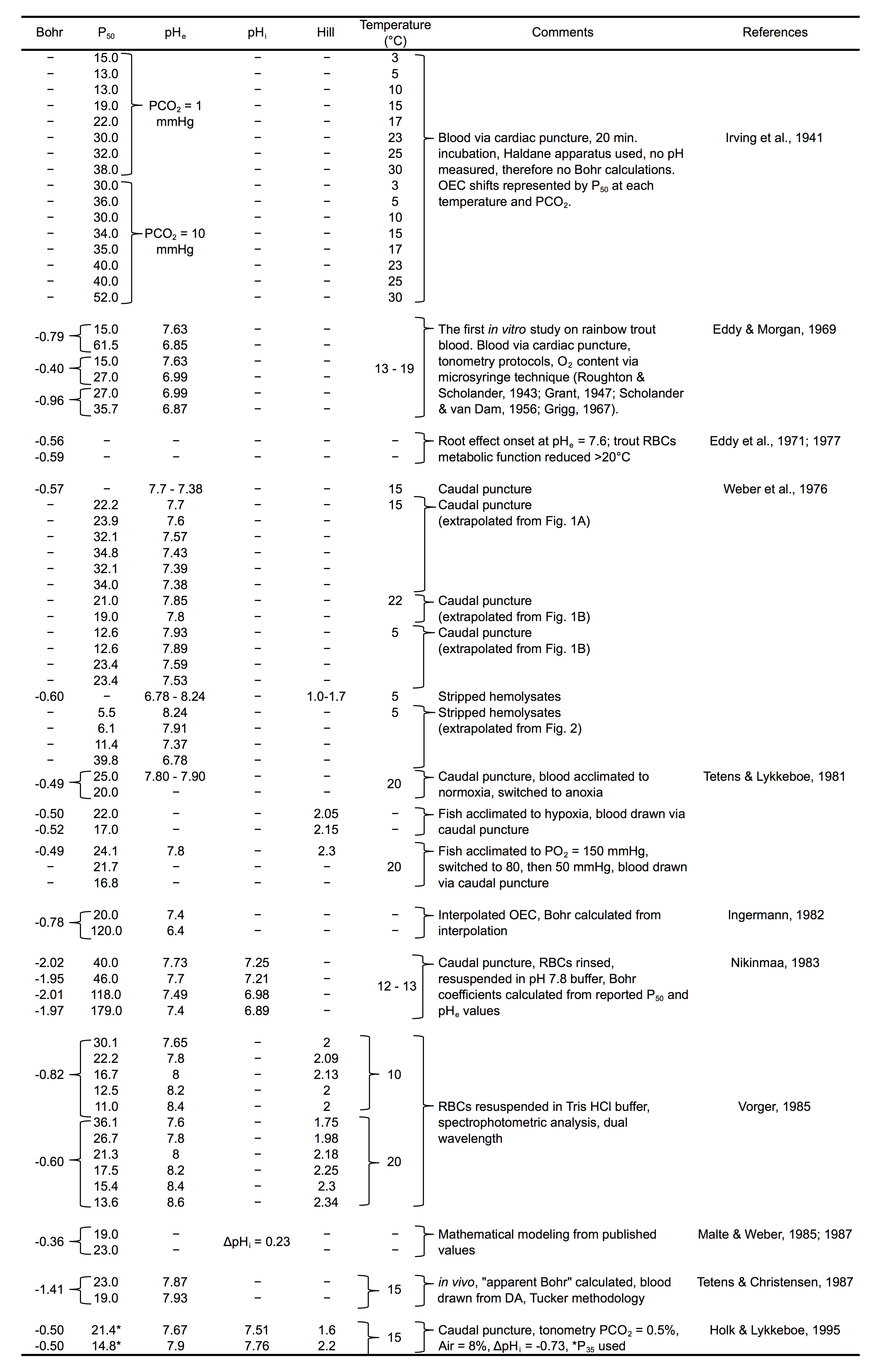
**

**S1 Fig. Hill plots for rainbow trout rinsed RBCs generated using tonometry.** Hill coefficients, n_H_ and P_50_ values derived from this plot are reported in Table 1. Data points represent means ±S.E.M.

**S2 Fig. Bohr coefficients (Φ) and the pH_e_ range over which they were calculated is plotted for the present study and those previously published.** If the Φ was not explicitly reported in the paper, it was extrapolated from the reported data. (Φ) = (ΔlogP_50_/ΔpH_e_). All values reported in this figure were determined *in vitro*, but for different preparations, including whole blood, RBC suspensions, isolated stripped Hb suspensions and using different conditions to generate the decrease in pH (hypoxia, CO_2_, HCl, or buffer suspensions) or from data sets built from an array of studies [41,54,57,60,63,64,69,70].

# Supplemental References

53. Irving L, Black EC, Safford V (1941) The influence of temperature upon the combination of oxygen with the blood of trout. Bio Bull 80: 1-17.

54. Eddy FB, Morgan RIG (1969) Some Effects of Carbon Dioxide on the Blood of Rainbow Trout *Salmo gairdneri* Richardson. J Fish Biol 1: 361-372.

55. Eddy FB (1971) Blood Gas Relationships in the Rainbow Trout Salmo Gairdneri. J Exp Biol 55: 695-711.

56. Eddy FB, Lomholt JP, Weber RE, Johansen K (1977) Blood respiratory properties of rainbow trout (Salmo gairdneri) kept in water of high CO2 tension. J Exp Biol 67: 37-47.

57. Weber RE, Wood SC, Lomholt JP (1976) Temperature acclimation and oxygen-binding properties of blood and multiple haemoglobins of rainbow trout. J Exp Biol 65: 333-345.

58. Tetens V, Lykkeboe G (1981) Blood respiratory properties of rainbow trout,Salmo gairdneri: Responses to hypoxia acclimation and anoxic incubation of blood in vitro. J Comp Physiol B 145: 117-125.

59. Ingerman RL, Terwilliger RC (1982) Presence and possible function of Root effect hemoglobins in fishes lacking functional swim bladders. J Exp Zool 220: 171-177.

60. Nikinmaa M (1983) Adrenergic regulation of haemoglobin oxygen affinity in rainbow trout red cells. J Comp Physiol B 152: 67-72.

61. Malte H, Weber RE (1985) A mathematical model for gas exchange in the fish gill based on non-linear blood gas equilibrium curves. Respiration Physiology 62.

62. Malte H, Weber RE (1985) A mathematical model for gas exchange in the fish gill based on non-linear blood gas equilibrium curves. Respir Physiol 62.

63. Tetens V, Christensen NJ (1987) β-adrenergic control of blood oxygen affinity in acutely hypoxia exposed rainbow trout. J Comp Physiol 157: 667-675.

64. Holk K, Lykkeboe G (1995) Catecholamine-induced changes in oxygen affinity of carp and trout blood. Respir Physiol 100: 55-62.

65. Roughton FJW, Scholander PF (1943) Micro gasometric estimation of the blood gases. J Biol Chem 148: 541-550.

66. Grant WC (1947) Determination of the oxygen capacity on 39.3 cu. mm. of blood. Proc Soc Exp Biol Med 66: 60.

67. Grigg GC (1967) Some respiratory properties of the blood of four species of Antarctic fishes. Comp Biochem Physiol 23.

68. Scholander PF, van Dam L (1956) Micro gasometric determination of oxygen in fish blood. J Cell Comp Physiol 48: 529-532.

69. Tetens V, Lykkeboe G (1985) Acute exposure of rainbow trout to mild and deep hypoxia: O_2_ affinity and O_2_ capacitance of arterial blood. Respir Physiol 61: 221-235.

70. Binotti I, Giovenco S, Giardina D, Antonini E, Brunori M, et al. (1971) Studies on the functional properties of fish haemoglobins. II. The oxygen equilibrium of the isolated haemoglobin components from trout blood. Ach Biochem Biophys 142: 274-280.
